# The use of filamentous hemagglutinin adhesin to detect immune responses to *Campylobacter hepaticus* infections in layer hens

**DOI:** 10.3389/fvets.2022.1082358

**Published:** 2022-12-21

**Authors:** Chithralekha Muralidharan, José A. Quinteros, Arif Anwar, Timothy B. Wilson, Peter C. Scott, Robert J. Moore, Thi Thu Hao Van

**Affiliations:** ^1^School of Science, Royal Melbourne Institute of Technology University, Bundoora, VIC, Australia; ^2^Scolexia Pty Ltd., Moonee Ponds, VIC, Australia

**Keywords:** *Campylobacter hepaticus*, Spotty Liver Disease, ELISA, filamentous hemagglutinin adhesion, sera, immunoassay

## Abstract

*Campylobacter hepaticus* is the aetiological agent of Spotty Liver Disease (SLD). SLD can cause significant production loss and mortalities among layer hens at and around peak of lay. We previously developed an enzyme linked immunosorbent assay (ELISA), SLD-ELISA1, to detect *C. hepaticus* specific antibodies from bird sera using *C. hepaticus* total proteins and sera pre-absorbed with *Campylobacter jejuni* proteins. The high specificity achieved with SLD-ELISA1 indicated the presence of *C. hepaticus* specific antibodies in sera of infected birds. However, some of the reagents used in SLD-ELISA1 are time consuming to prepare and difficult to quality control. This understanding led to the search for *C. hepaticus* specific immunogenic proteins that could be used in recombinant forms as antibody capture antigens in immunoassay design. In this study, an immunoproteomic approach that combined bioinformatics analysis, western blotting, and LC MS/MS protein profiling was used, and a fragment of filamentous hemagglutinin adhesin (FHA), FHA_1,628−1,899_ with *C. hepaticus* specific antigenicity was identified. Recombinant FHA_1,628−1,899_ was used as antigen coating on ELISA plates to capture FHA_1,628−1,899_ specific antibodies in sera of infected birds. SLD-ELISA2, based on the purified recombinant FHA fragment, is more user-friendly and standardizable than SLD-ELISA1 for screening antibody responses to *C. hepaticus* exposure in hens. This study is the first report of the use of FHA from a *Campylobacter* species in immunoassays, and it also opens future research directions to investigate the role of FHA in *C. hepaticus* pathogenesis and its effectiveness as a vaccine candidate.

## Introduction

*Campylobacter hepaticus*, the causative agent of Spotty Liver Disease (SLD), is closely related to *Campylobacter jejuni* and *Campylobacter coli* bacteria that are common causes of gastrointestinal diseases in humans. The nucleotide identity of *C. hepaticus* with *C. jejuni* and *C. coli* is 83 and 80%, respectively, whereas the average nucleotide identity shared with other campylobacters is about 75% (1). Approximately, 70% of the genes in *C. hepaticus* are conserved in all three species (2). Despite the close relatedness at the genetic level, disease presentation of *C. hepaticus* is very different from that of *C. jejuni* and *C. coli*. The latter often infect chickens in the first few weeks after hatching and colonize their gastrointestinal tracts without causing evident clinical signs (3). In contrast, *C. hepaticus* infection causes a serious illness in layer birds that affects egg production and in certain cases, results in sudden death (4–6). Genes present in other campylobacters, that may have a role in pathogenesis, such as cytolethal distending toxin (cdt) genes, are absent in *C. hepaticus* (2).

A comparative genomics study of *C. hepaticus* isolates from the UK, including comparisons to the genomic sequences of 24 other species of *Campylobacter*, identified a 140 kb reduction in genome size, with around 144 fewer genes and a lower GC content in *C. hepaticus*. Most gene reduction was reported in the subsystem containing genes for iron acquisition and metabolism. The authors hypothesized that the loss of the genes may have been an adaptive mechanism of *C. hepaticus* to thrive in the iron rich niche of the chicken liver (7). Another genomics and comparative transcriptomic analysis of Australian *C. hepaticus* isolates identified 213 putative virulence genes in *C. hepaticus*, out of which 46 were upregulated in *C. hepaticus* recovered from the bile of infected birds (*in vivo*) compared to lab grown isolates (*in vitro*) (2). However, these studies did not provide any information on unique immunogenic proteins in *C. hepaticus* that could differentiate it from other campylobacters.

A previously developed *C. hepaticus* specific enzyme linked immunosorbent assay (ELISA), referred as SLD-ELISA1, enabled the detection of *C. hepaticus* specific antibodies present in the sera of infected birds. It used *C. hepaticus* total proteins as antigen coating on ELISA plates and required a pre-absorption step using *C. jejuni* proteins. A significant proportion of chickens across all production systems are infected with *C. jejuni* and/or *C. coli* and have antibodies to those bacteria (3, 8, 9). It is therefore important to be able to differentiate between antibody responses to these ubiquitous infections and *C. hepaticus* infection. Pre-absorption removes cross-reactive antibodies from bird sera that would otherwise have bound to *C. hepaticus* proteins and resulted in false positive readings. The successful differentiation of SLD positive and negative sera samples using SLD-ELISA1 indicated the presence of *C. hepaticus* specific immunogenic proteins that are not cross reactive with *C. jejuni* antibodies commonly present in bird sera (10). Although the assay was highly specific and sensitive, there were a few shortcomings that made it less than ideal for large scale use. The inherent variability of protein coating requires enhanced standardization procedures to prevent batch to batch variations of *C. hepaticus* total protein preparations. The pre-absorption step required additional time, lab consumables, and *C. jejuni* total protein preparation. This was inconvenient, labor intensive, and time-consuming, especially when working with a large number of samples, and limited the number of samples that could be processed at a given time. An ELISA that uses a specific purified *C. hepaticus* recombinant protein as capture antigen could eliminate these disadvantages.

In the present study, an immunoproteomic approach was used, that combined bioinformatics analysis, western blotting, and mass spectroscopy-based protein profiling of *C. hepaticus* proteins to identify *C. hepaticus* specific immunogenic proteins. The purified protein was then used as the antigen coating on ELISA plates to detect *C. hepaticus* specific antibodies present in the sera of infected birds.

## Materials and methods

### Prediction of *C. hepaticus* specific immunogenic proteins using bioinformatics analysis

**Figure 1:**
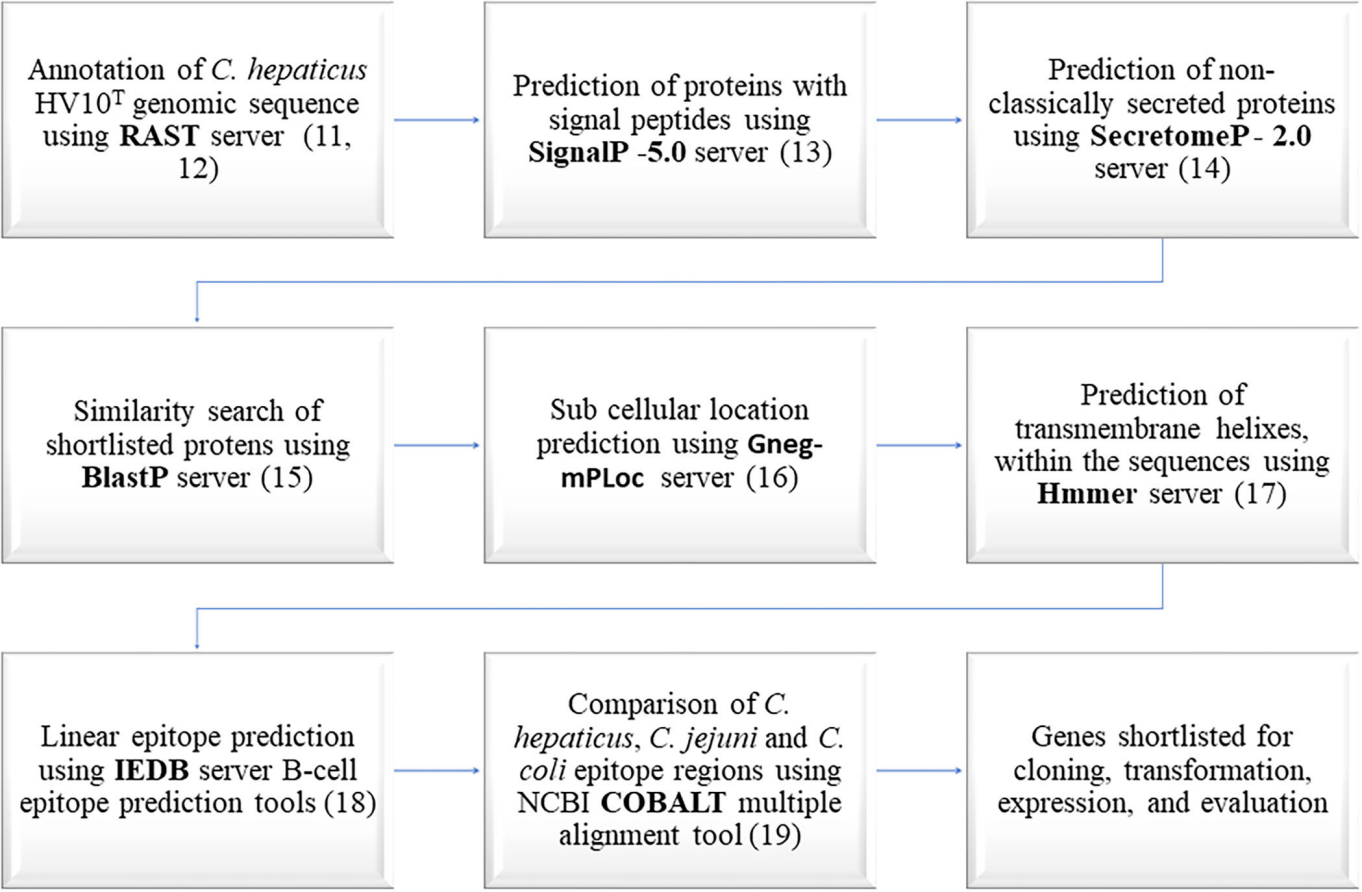
Bioinformatics workflow used in the prediction of *C. hepaticus* specific immunogenic proteins.

The whole genome sequences of 14 Australian *C. hepaticus* strains were annotated using the Rapid Annotation using Subsystem Technology (RAST) server (11, 12) to identify predicted proteins encoded by the *C. hepaticus* core genome. Annotated protein sequences from *C. hepaticus* type strain HV10^T^ were used in all bioinformatics analysis (1). The bioinformatics workflow used to predict *C. hepaticus* specific immunogenic proteins is outlined in Figure 1. The first step was to shortlist surface exposed and secreted proteins from the predicted *C. hepaticus* proteome using SignalP (13) and SecretomeP (14) servers because these proteins have the highest likelihood of being immunogenic.

The amino acid sequences of *C. hepaticus* proteins predicted as non-classically secreted proteins or proteins with signal peptides were compared with protein sequences in the National Center for Biotechnology Information (NCBI) databases using the BlastP server (15). Proteins that shared 85% or above identity with *C. jejuni* and *C. coli* proteins were removed from further analysis as the chances of them being useful in identifying a specific *C. hepaticus* driven immune response were very low. The subcellular location of the proteins were predicted using Gneg-mPLoc server (16). HMMER server was used to predict the presence of transmembrane helixes and disordered regions within the amino acid sequences (17). The epitopes present within the amino acid sequences were predicted using the Immune Epitope Database (IEDB) server (18). The constraint-based alignment tool (COBALT) (19) was used to align the shortlisted proteins with one each of *C. coli* and *C. jejuni* homologous protein sequences that appeared as top hits in the BlastP search. The amino acid sequences within the epitope regions of *C. hepaticus* proteins, predicted using the IEDB server, were manually compared with homologous sequences from *C. coli* and *C. jejuni* aligned using COBALT, to identify *C. hepaticus* proteins with the least similar epitope regions. Proteins with high antigenicity scores and significant amino acid differences within the epitope regions of the *C. hepaticus* sequence compared to *C. jejuni* and *C. coli* sequences were shortlisted for cloning and expression studies. The conservation of shortlisted protein encoding genes in all 14 *C. hepaticus* strains was also checked.

### Identification of *C. hepaticus* specific immunogenic proteins by western blotting

*C. hepaticus* HV10^T^ (NCBI Accession number CP031611), *C. jejuni* 81116 (NCTC 11828) and *C. coli* (NCTC 11366) total protein extracts (TPE) were prepared as described previously (10). Protein concentration of the TPEs were determined as a measure of optical densities (OD) at 280 nm on a Nanodrop microvolume spectrophotometer (Thermo Fisher Scientific). The protein preparations (OD 1) were run on 4–20% Mini-PROTEAN^®^ TGX™ precast gels (Bio-Rad). Protein bands in gels were stained using Coomassie brilliant blue R-250 (Sigma) or transferred to polyvinylidene difluoride (PVDF) membranes using iBlot™ Transfer Stack, PVDF, mini packs and iBlot^®^ Dry Blotting System (Thermo Fisher Scientific) to perform western blotting. Blotted PVDF membranes were incubated for an hour in blocking solution [5% skim milk powder diluted in phosphate buffer saline with 0.05% Tween 20 (PBST)]. Blocked membranes were probed using one in 250 dilution of bird sera diluted in blocking solution (normal primary antibody solution) or pre-absorbed primary antibody solution for an hour, washed three times with PBST, followed by 1 h incubation with one in a thousand dilution of goat anti-chicken IgY-HRP (Thermo Fisher Scientific) diluted in blocking solution. The membrane was washed three times with PBST and developed using 3,3′,5,5′-tetramethylbenzidine (TMB) substrate (Invitrogen). Pre-absorbed primary antibody solution is the supernatant obtained after spinning down the normal primary antibody solution incubated overnight with 50 μL each of OD 4 *C. jejuni* and *C. coli* TPEs. Protein bands from *C. hepaticus, C. jejuni*, and *C. coli* TPE lanes on PVDF membranes probed using normal and pre-absorbed primary antibody solutions were compared and the unique bands that are present only in *C. hepaticus* were noted. Two-dimensional (2D) gel electrophoresis was also performed using Zoom IPG runner system and reagents to resolve the bands further as per manufacturers protocol (Thermo Fisher Scientific).

### Protein identification using liquid chromatography electrospray ionization tandem mass spectrometry (LC ESI MS/MS)

Proteins bands in Coomassie stained gels that corresponded to *C. hepaticus* specific bands identified by western blotting were excised and trypsin digested. Proteins present within the bands were identified by LC ESI MS/MS (Monash Proteomics and Metabolomics facility, Melbourne, Australia). A Dionex Ultimate 3000 RSLCnano Nano LC System and QExactive HF Mass spectrometer with Acclaim PepMap RSLC (75 μm × 50 cm, nanoViper, C18, 2 μm, 100Å) analytical column and Acclaim PepMap 100 (100 μm × 2 cm, nanoViper, C18, 5 μm, 100Å) trap column was used (Thermo Fisher Scientific). Data analysis was performed using the Byonic search engine (ProteinMetrics) by comparing experimentally obtained peptide spectra with theoretical peptide spectra generated from a RAST annotated *C. hepaticus* HV10^T^ protein database and SwissProt database separately. The ten top ranked proteins obtained from the *C. hepaticus* HV10^T^ proteins database comparison was inspected for potential immunogenic proteins.

### Determination of immunogenicity of shortlisted proteins by cloning, expression, and western blotting

Protein encoding genes that were shortlisted though the bioinformatics and western blotting/LC ESI MS/MS analysis were synthesized and cloned in pET-28(+)-TEV vector (GenScript gene synthesis service). For smaller proteins, the whole coding sequence was used but for larger proteins, a fragment of the coding sequence containing several IEDB predicted immunogenic epitopes was used. The genes were codon optimized for expression in *Escherichia coli*. The plasmids containing genes of interest were transformed into chemically competent *E. coli* BL21 (DE3) cells prepared in-house (20) or obtained from New England Biolabs for expression by a heat-shock induction method based on the manufacturer's protocol. Protein expression from three to five clones was carried out at various temperatures (16, 22, 30 and 37°C), incubation times (4 h to overnight) and induced using 0.05–5 mM Isopropyl β-d-1-thiogalactopyranoside (IPTG) to optimize the expression. *E. coli* cells were lysed by sonication and checked for expression by Coomassie staining and anti-his tag western blotting. The immunogenicity of expressed proteins was determined by western blotting using pooled SLD positive and SLD negative bird sera. Expressed proteins that showed immunogenicity only with the pooled SLD positive sera were further tested for *C. hepaticus* specific immunogenicity using individual SLD positive and SLD negative bird sera. The recombinant protein recognized by the majority of SLD positive sera and fewest SLD negative sera was purified on immobilized metal affinity chromatography (IMAC) polypropylene columns (Sigma) packed with nickel-nitriloacetic acid (Ni-NTA) agarose (Thermo Fisher Scientific). Purification was performed under native conditions with elution using a 50–500 mM imidazole (Sigma) step gradient. The elutes that contained target proteins were pooled, and buffer exchanged with PBS to remove imidazole using Amicon 10 kDa ultracentrifuge filter units (Merck).

### Development of ELISA using purified recombinant protein, SLD-ELISA2

A total of 115 sera samples were used for the development and validation of SLD-ELISA2, including sera samples from 48 experimentally infected birds, 10 naturally infected birds, and 57 negative control birds. Sera collection and processing was done as described previously (10). The sera samples from naturally infected birds were collected in the course of normal veterinary care for flocks. In the experimental infection trial (to be fully reported elsewhere), layer hens entering the peak lay period were infected with *C. hepaticus* HV10^T^ using the method of Van et al. (21) and then monitored for an extended period of time. Sera samples from four different experimental groups were included in this study. Each group had 12 birds and were sampled once (3, 6, 9, or 12 weeks post-challenge). Hence, 48 sera samples were obtained from experimentally infected birds. *C. hepaticus* was recovered in bile cultures from 92% of the experimentally infected birds, which confirmed the SLD status of birds. The experimental infection trials at the Scolexia facility were approved by Agriculture Victoria, Wildlife and Small Institutions Animal Ethics Committee (Approval number 33.21).

All sera samples were initially assayed using SLD-ELISA1 to confirm their SLD immune response status. Sera from two naturally infected birds, two experimentally infected birds and three negative control birds were used for the optimisation of SLD-ELISA2. These seven sera samples represent the strong and weak SLD-positive and negative samples identified in SLD-ELISA1 and was included in the optimisation of SLD-ELISA2 to take into account the bird-to-bird variation in immune responses that are reflected in the absorbance range of true positive and negative samples. Purified recombinant protein at 0.25, 0.5, 1.0, and 2.0 μg/ml diluted in PBS was tested to determine the optimal protein concentration for coating ELISA plates. Bird sera at one in 500, 1,000, 2,000, and 4,000 dilutions were tested to identify the optimal sera dilution that could differentiate SLD positive and SLD negative sera samples. The SLD-ELISA2 protocol was as follows. Nunc Maxisorp 96 well plates (Thermo Fisher Scientific) were coated overnight at 4°C with the recombinant protein (50 μl/well). The wells were washed with PBST and incubated with 200 μl of blocking solution (5% skim milk powder in PBST) for 2 h at room temperature (RT). The wells were washed again and incubated with 100 μl of bird sera diluted in blocking solution for 2 h at RT. The wells were then washed four times and incubated with 100 μl of goat anti-chicken IgY-HRP (Thermo Fisher Scientific) secondary antibody (1:2000 dilution in blocking buffer) for an hour at RT. TMB substrate (Invitrogen) was used for chromogenic detection (50 μl/well). The absorbance was read at 450 nm on a POLAR star Omega Plate Reader Spectrophotometer (BMG LABTECH) following the addition of 50 μl of stopping solution (2 M sulphuric acid). The cut-off value for the assay was calculated as the absorbance mean plus two standard deviations (SD) of all 57 negative control samples included in this study (22). Assay sensitivity and specificity was calculated as described previously (23). Statistical analysis was carried out using GraphPad Prism (9.0.2) software (San Diego, CA, USA). A one-way ANOVA test was performed assuming equal Gaussian distribution of residuals and equal standard deviations with 95% confidence interval. Multiple comparison was performed by comparing the mean of each group with the mean of every other group.

### SLD-ELISA2 assay precision

All samples were assayed in triplicates. Serum from a naturally infected bird that showed high absorbance reading (positive control) and a negative control sample were included in triplicates as quality control samples in all assay plates to ensure the inter-assay variation was within the acceptable limit of 15%. The coefficients of variation (CV) of all SLD positive samples were calculated as described previously to determine the intra-assay precision (24, 25). The CV of the positive control sample assayed in six replicates on 12 different days was used to determine the inter-assay precision.

## Results

### Prediction of *C. hepaticus* specific immunogenic proteins using bioinformatics analysis

The RAST server predicted 1,521 protein encoding genes (PEGs) in the *C. hepaticus* HV10^T^ genome, of which 167 were predicted to contain signal peptides, and 85 were predicted as non-classically secreted proteins. Among those 252 proteins, 142 proteins that shared <85% amino acid identity with *C. jejuni* and *C. coli* homologs were shortlisted for further analysis. From 142 candidates, 19 proteins that had high IEDB predicted antigenicity scores, predicted extracellular or outer membrane location, and significant amino acid differences of the *C. hepaticus* sequence compared to *C. jejuni* and *C. coli* sequences within the epitope regions predicted using IEDB server were shortlisted for cloning and expression studies. PEGS 10, 610, and 1,126 were included despite their predicted periplasm, cytoplasm, or cell inner membrane location because of their lower identity with *C. jejuni* and *C. coli* homologs and high antigenicity scores.

PEGs shortlisted for cloning and expression studies are presented in Table 1. The length of nucleotide sequence in each PEG used for gene synthesis and cloning was determined based on the size of the protein, presence of signal peptides, or transmembrane helices predicted within the corresponding protein sequences. Proteins that are larger than 800 amino acids can be difficult to express in *E. coli*. Therefore, amino acid regions containing multiple epitopes that had lowest identity to equivalent *C. jejuni* and *C. coli* proteins in PEGs 508, 1,027, and 1,316, spanning 311–523, 781–1,074 and 1,628–1,899 amino acid residues, respectively, were selected for protein expression. Signal peptide sequences from PEGs 140, 407, 1,296, 1,335, and 1,488 were omitted from gene synthesis as they could interfere with protein localization after expression. A transmembrane sequence spanning 27 amino acid residues at the N-terminal region of PEG 571 was also omitted from gene synthesis and cloning as it could pose difficulties in expression. Complete sequences of PEGs 10, 433, 486, 493, 610, 734, 918, 1,021, 1,033, and 1,126 were used for gene synthesis and cloning.

**Table 1:** PEGs shortlisted for cloning and expression using bioinformatics tools.

**PEG No**.	**Annotation**	**Protein class**	**Identity to *C. jejuni* (%)**	**Identity to *C. coli* (%)**	**Predicted cellular locations**
10	Oligopeptide ABC transporter, periplasmic oligopeptide-binding protein OppA (TC 3.A.1.5.1)	SP (1–18)	48.83	24.70	Periplasm
140	Major outer membrane protein (MOMP)	SP (1–22)	77.08	75.06	Cell outer membrane
407	Surface-exposed lipoprotein JlpA	LIPO (1–17)	58.33	58.60	Extracellular
433	FIG00638667: hypothetical protein	SP (1–16)	61.48	60.25	Cell inner membrane, Extracellular
486	Flagellar hook-associated protein FlgL	NSP (0.955)	81.89	78.91	Extracellular
493	FIG00638667: hypothetical protein	SP (1–19)	71.43	70.95	Cell inner membrane, Extracellular
508	FIG00469420: hypothetical protein	NSP (0.927)	64.00	64.85	Cell inner membrane, Extracellular
571	Probable periplasmic protein Cj0776c	NSP (0.602)	61.19	60.36	Extracellular
610	Glycerophosphoryl diester phosphodiesterase, periplasmic (EC 3.1.4.46)	SP (1–17)	68.42	68.42	Cytoplasm
734	FIG00545237: hypothetical protein	LIPO (1–19)	48.84	47.09	Extracellular
918	Possible lipoprotein	NSP (0.885)	31.31	32.83	Extracellular
1,021	FIG00469721: hypothetical protein	NSP (0.873)	74.34	75.22	Extracellular
1,027	Possible lipoprotein	SP (1–39)	36.26	37.60	Cell outer membrane, Extracellular
1,033	FIG00638667: hypothetical protein	SP (1–19)	59.36	60.16	Extracellular
1,126	Allophanate hydrolase 2 subunit 2 (EC 3.5.1.54)	NSP (0.735)	30.35	28.95	Cell inner membrane
1,296	Probable lipoprotein	LIPO (1–16)	72.08	66.01	Cell inner membrane, Cell outer membrane
1,316	Filamentous hemagglutinin/adhesin	SP (1–28)	71.72	56.79	Cell outer membrane, Extracellular
1,335	Haemin uptake system outer membrane receptor	SP (1–23)	75.07	74.54	Cell outer membrane
1,488	FIG00638667: hypothetical protein	SP (1–16)	69.34	68.98	Cell inner membrane, Cell outer membrane

SP (region), standard signal peptide; LIPO (region), lipoprotein signal peptide; NSP (score), non-classically secreted protein.

### Identification of *C. hepaticus* specific immunogenic proteins by western blotting and LC-MS/MS protein profiling

**Figure 2:**
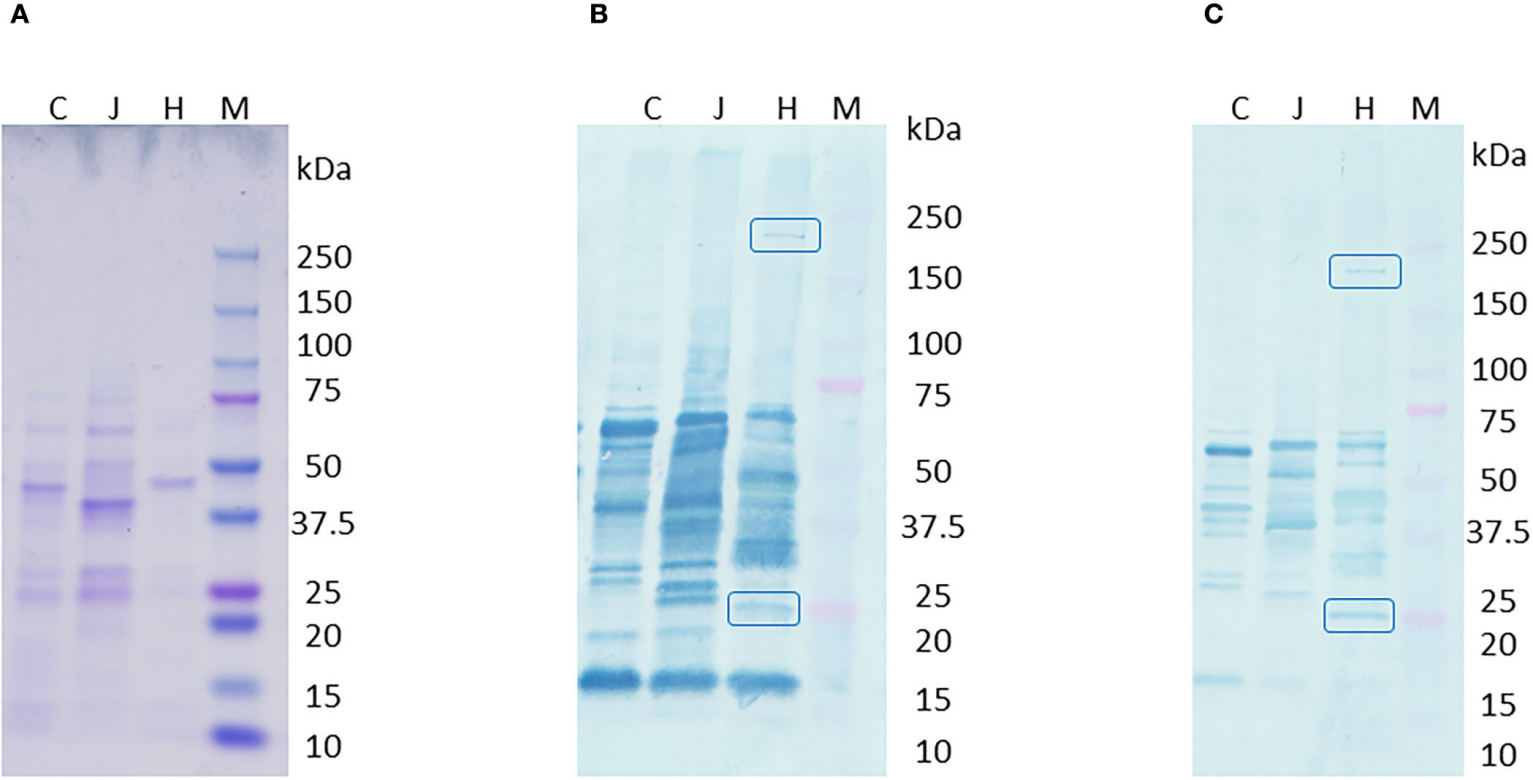
Western blot of *C. hepaticus, C. jejuni*, and *C. coli* total proteins probed with pooled SLD positive sera. **(A)** Coomassie stained gel, **(B)** PVDF membrane probed with SLD positive pooled sera that showed specific bands at 220 and 25 kDa regions, and **(C)** PVDF membrane probed with pre-absorbed SLD positive pooled sera that still showed the specific bands at 220 and 25 kDa. Lane M–Precision plus protein ladder (Bio-Rad). Total protein profiles of *C. hepaticus*–H, *C. jejuni*–J, and *C. coli*–C.

Western blot of *C. hepaticus, C. jejuni*, and *C. coli* total proteins probed with pooled SLD positive sera showed bands at 220 and 25 kDa that were present only in *C. hepaticus*, as shown in Figure 2. Western blotting using pre-absorbed SLD positive pooled sera also retained the two bands and confirmed their specificity. Therefore, the bands at 220 and 25 kDa were analyzed using LC MS/MS protein profiling to determine the identity of immunogenic proteins present within the bands. A mixture of proteins was identified within each band, which is usual for gel bands. The top band (220 kDa) matched well with a protein annotated as filamentous hemagglutinin adhesin (FHA, PEG 1,316). It is the largest protein encoded in *C. hepaticus* genome and is composed of 1,899 amino acids. The proteins in the successive rankings were RNA polymerase beta subunit proteins and cytoplasmic proteins which also matched to *C. jejuni* proteins in the SwissProt database search. Hence, they were not considered likely to be immunogenic proteins. Moreover, no bands were observed around 220 kDa region in *C. jejuni* or *C. coli* protein lanes on western blots. The band at 25 kDa produced major outer membrane protein (MOMP, PEG 140) as the top match. Lower ranked proteins were cytoplasmic and non-immunogenic proteins. The top ranked proteins, FHA and MOMP, that matched with the two *C. hepaticus* specific bands at 220 and 25 kDa, encoded by PEGs 1,316 and 140 were also identified as potential candidates for protein expression studies using bioinformatics analysis.

### Determination of immunogenicity of shortlisted proteins by cloning, expression, and western blotting

**Figure 3:**
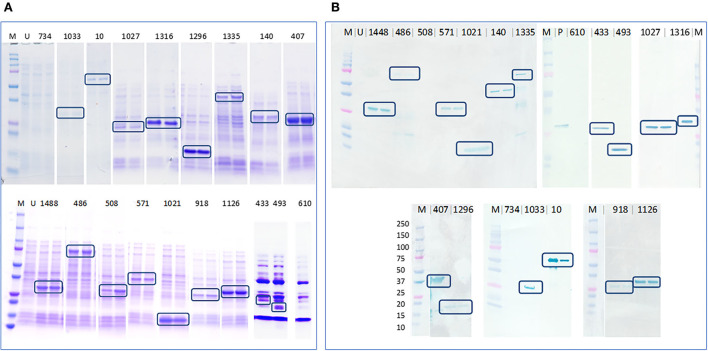
Determination of protein expression in *E. coli* cell lysates by Coomassie staining and anti-his western blotting. **(A)** Coomassie stained gel containing expressed *E. coli* cell lysates that confirmed the expression of 17 out of 19 proteins, highlighted in blue boxes and **(B)** PVDF membrane blotted with expressed *E. coli* cell lysates probed with anti-his antibody that confirmed the expression of 16 out of 19 proteins, highlighted in blue boxes. M–Precision plus protein ladder (Bio-Rad), U–uninduced *E. coli* BL21 (DE3) cell lysate, P–an unrelated his-tagged protein used as a positive control.

Seventeen out of the 19 cloned genes/ gene fragments were successfully expressed in *E. coli*. The Coomassie stained gel bands and anti-his western blots that recognized the expressed proteins are shown in Figure 3. PEGs 610 and 734 were not expressed despite using different temperatures, IPTG concentrations, extended time, and host strains for protein expression. Recombinant protein expressed by PEG 508 was not detected in the anti-his western blotting even though the expressed protein was seen as distinct band in the Coomassie stained gel indicating the possibility of poor his tag accessibility for anti-his antibody binding.

**Figure 4:**
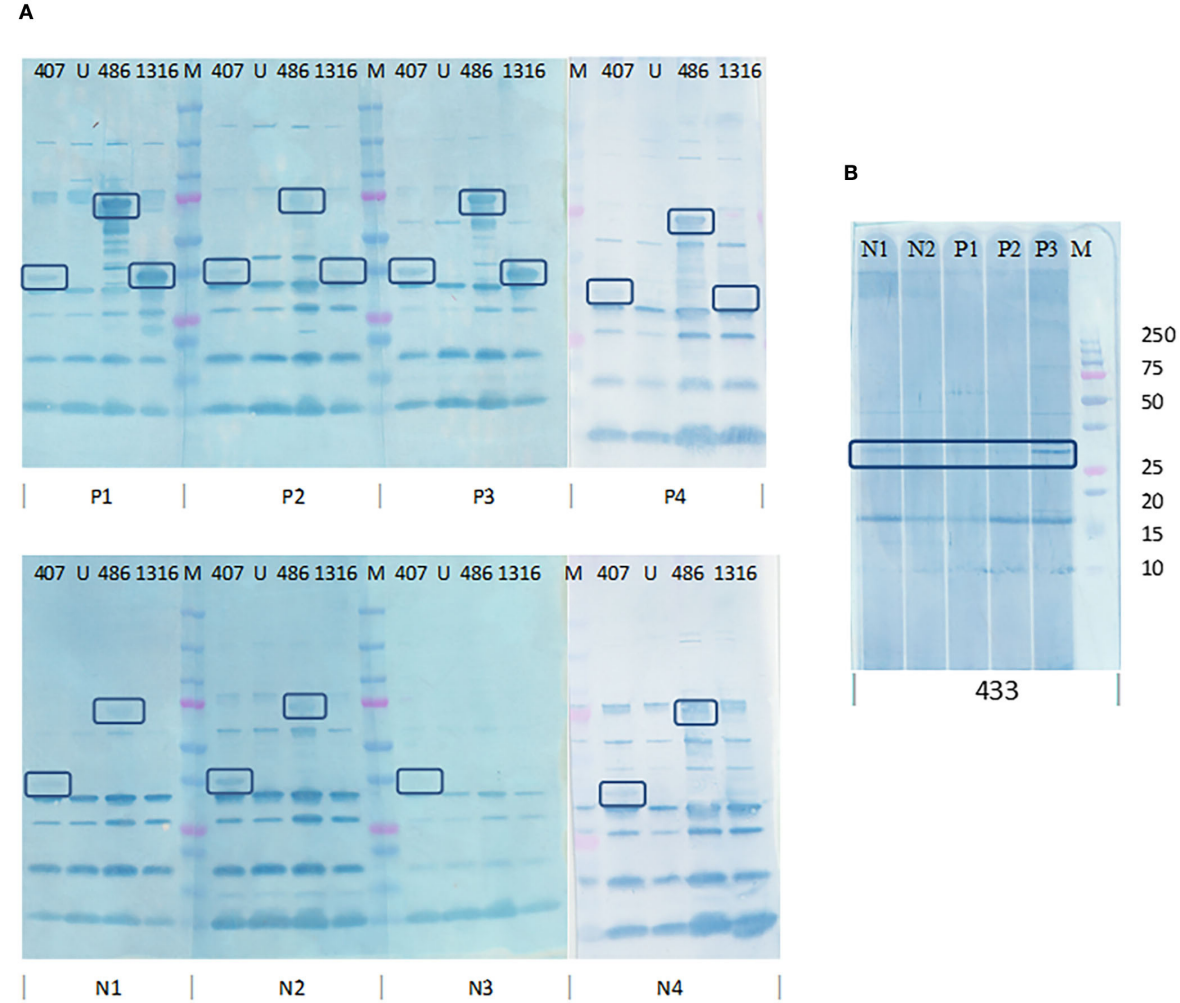
Determination of *C. hepaticus* specific immunogenicity of the recombinant proteins coded by PEGs 407, 433, 486, and 1,316 by western blotting using individual SLD positive and negative sera. **(A)** PVDF membrane blotted with *E. coli* cell lysates (PEGs 407, 486, and 1,316) and probed with sera from four SLD positive birds, P1, P2, P3, and P4 and four negative control birds, N1, N2, N3, and N4. **(B)** PVDF membrane blotted with *E. coli* cell lysate (PEG 433) and probed with sera from three SLD positive birds, P1, P2, and P3, and two negative control birds, N1 and N2. Only the recombinant protein encoded by PEG 1,316 showed *C. hepaticus* specific immunogenicity as it was recognized by SLD positive sera and not by the negative control sera. The recombinant proteins encoded by PEGs 433, 407, and 486 cross reacted with antibodies in several SLD negative sera. M–Precision plus protein ladder (Bio-Rad).

PVDF membranes blotted with *E. coli* cell lysates after expression were probed using pooled SLD positive sera to determine the immunogenicity of the 17 expressed recombinant proteins. The recombinant proteins encoded by PEGs 407, 433, 486, and 1,316 were recognized by the antibodies in pooled sera. The four proteins showed distinct bands of the expected size on western blots probed using pooled SLD positive sera, Coomassie staining and anti-his western blotting, and each was absent in the uninduced *E. coli* cell lysates. However, when probed using individual SLD positive and SLD negative sera, only the recombinant protein encoded by PEG 1,316, a fragment of FHA, showed *C. hepaticus* specific immunogenicity (Figure 4). The recombinant protein encoded by PEG 407 cross-reacted with all four negative control sera, the recombinant protein encoded by PEG 486 cross-reacted with three of the four negative control sera and the recombinant protein encoded by PEG 433 cross-reacted with both negative control sera assayed suggesting that these proteins are not specific to *C. hepaticus*. Therefore, the recombinant FHA fragment produced by *E. coli* harboring PEG 1,316 gene fragment was used in the development of SLD-ELISA2. The recombinant FHA fragment was named as FHA_1,628−1, 899_, to indicate the length and position of amino acid residues within the native FHA protein that were included in the recombinant protein. The optimal expression conditions to produce recombinant FHA_1,628−1,899_ was 4 h of incubation after induction with 0.1 mM IPTG at 37°C.

### Development of SLD-ELISA2 using purified recombinant FHA_1,628–1,899_

**Figure 5:**
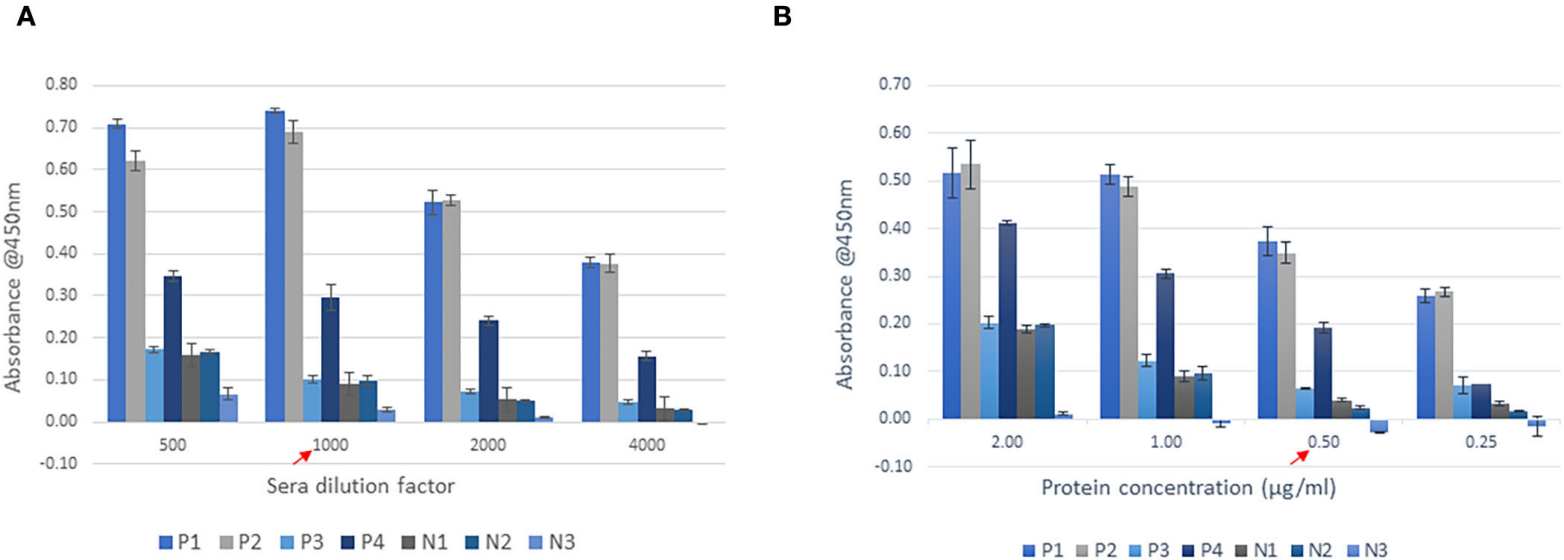
Optimisation of SLD-ELISA2 parameters. **(A)** Sera dilution and **(B)** Protein coating. The red arrows indicate the optimal values.

Purified recombinant FHA_1,628−1,899_ was used as the coating protein on ELISA plates. The optimal antigen coating concentration on ELISA plates was identified as 0.5 μg of purified protein/ml as the difference in absorbance values of the positive control P3, and negative controls N1 and N2 were very low in 1.0 and 2.0 μg/ml coating. The difference between positive and negative samples were furthermore in 0.25 μg/ml coating, nevertheless, the absorbance of SLD-positive samples also reduced. Therefore, 0.5 μg/ml was chosen as the optimal coating concentration to improve the assay sensitivity (minimize the chance of weak positives being categorized as negatives due to low absorbance) and assay specificity (reduce the absorbance from negative samples). One in a thousand dilution of sera was determined to be optimal for distinguishing SLD positive and SLD negative samples as the absorbance of positive samples in 1,000-fold dilution was close to that from 500-fold dilution, and it dropped at 2,000-fold dilution. The absorbance from negative controls was steadily decreasing with dilution (Figure 5).

**Figure 6:**
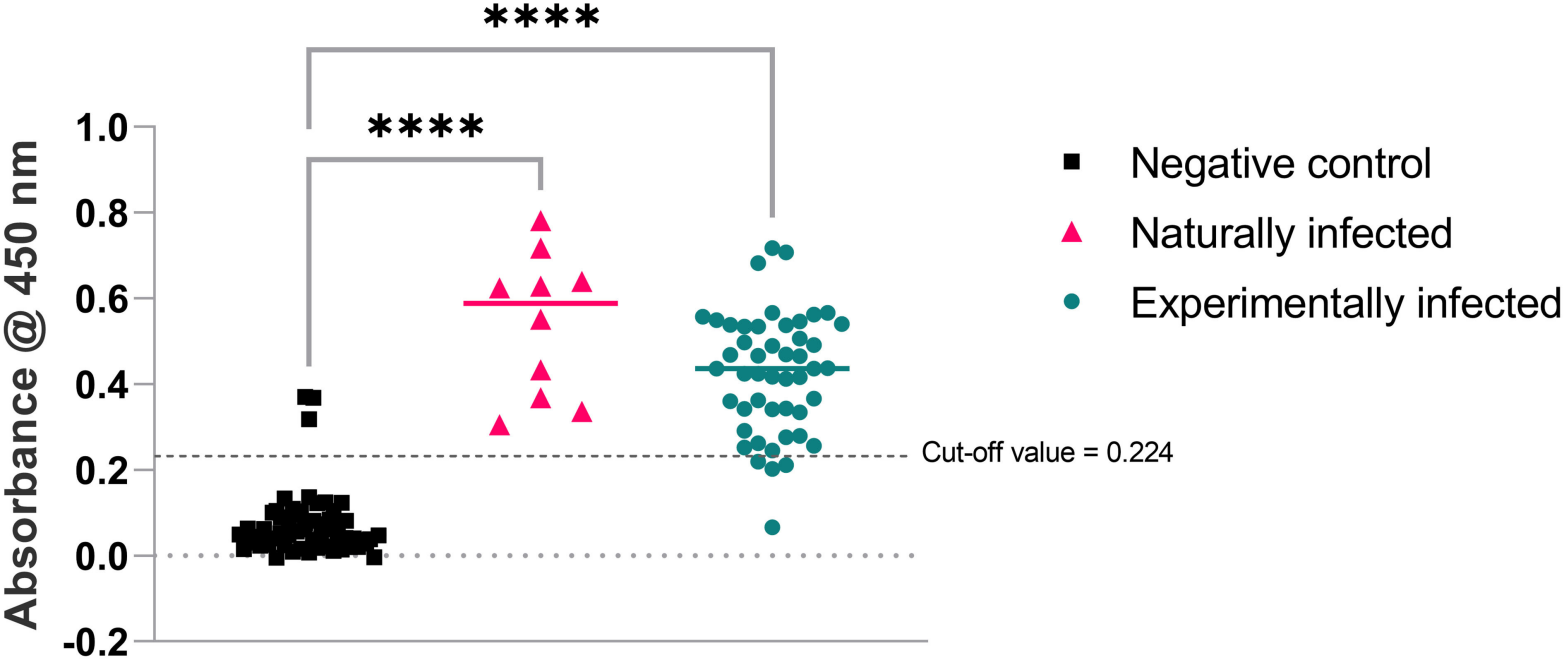
Absorbance produced by anti-FHA antibodies present in the bird sera used in SLD-ELISA2. Ordinary one-way ANOVA pairwise comparison results were highly significant with ****p < 0.0001 for both naturally and experimentally infected groups compared to the negative control group.

The cut-off value for the assay was calculated as 0.224 (mean + 2SD of 57 negative control samples used in this study). The sera samples used to develop and test SLD-ELISA2 from naturally and experimentally infected birds were all but one positive in SLD-ELISA1 (98% sensitivity). The sera samples from all negative control birds were negative in SLD-ELISA1 (100% specificity). SLD-ELISA2 results were congruent with SLD-ELISA1 in 94% (108/115) of the samples. These negative control birds were shown to contain either or both of *C. jejuni* and *C. coli* DNA (10). Therefore, SLD-ELISA2 negative results from those birds confirms that the anti-*C. jejuni* and *C. coli* antibodies are not cross-reactive to FHA_1,628−1,899_. The 10 sera samples from the naturally infected group and 44 of 48 samples from the experimentally infected group had absorbance values well above the cut off value 0.224, resulting in 93.1% assay sensitivity (Figure 6). The assay specificity was 94.73% with the absorbance values of all but three sera samples from the negative control group below the cut-off value. Statistical analysis using one way ANOVA confirmed that the absorbance values of the naturally and experimentally infected groups were significantly different to the negative control group with a *p* < 0.0001 (Figure 6).

### SLD-ELISA2 assay precision

Intra-assay precision of SLD-ELISA2 ranged between 1 and 9% with an average coefficient of variation (CV) of 3%. The inter-assay variation of the positive control sample assayed over 12 different days in six replicates was 13.7% with a mean absorbance value 0.706 and standard deviation 0.097. Absorbance value corrections was made for samples assayed in each plate by multiplying the absorbance values of all samples by the ratio (0.706/actual absorbance of positive control obtained in that ELISA plate), to standardize the absorbance value of positive control in each assay plate to 0.706.

## Discussion

Filamentous hemagglutinin adhesin has been reported to be a major virulence factor in several human and animal pathogens, including *Acinetobacter baumannii, Bordetella pertussis, Bordetella bronchiseptica, Moraxella catarrhalis*, and *Pseudomonas fluorescens* (26–30). The main function of FHA is to aid in the attachment of pathogens to host cells. FHA from *Bordetella pertussis* has been used in acellular pertussis vaccines because of its immunogenicity (30, 31). The role of FHA in cell adhesion has also been reported in several *C*. *jejuni* and *C. coli* strains (32–34). However, the identity of *C. hepaticus* FHA with FHA present in other *Campylobacter* species was <72%. The identity of the immunogenic fragment identified in this study, FHA_1,628−1,899_ was further low (<64%). This variability in amino acid residues could be the reason for *C. hepaticus* specific immunogenicity of FHA_1,628−1,899_. The present study is the first report of the use of an FHA from a *Campylobacter* species in immunological assays.

Immunoproteomic methods have previously been used for the identification of immunogenic proteins from several pathogens (35–39). FHA_1,628−1,899_ was identified as a *C. hepaticus* specific immunogenic protein from a group of 19 proteins that were progressively shortlisted from 1,518 *C. hepaticus* PEGs after extensive bioinformatic analysis. Even though three other recombinant proteins annotated as surface-exposed lipoprotein JlpA, flagellar hook-associated protein FlgL and a hypothetical protein showed immunogenicity, they were cross-reactive with antibodies present in sera of birds from the negative control group, presumably indicating that antibodies to other campylobacter infections, such as *C. jejuni* and *C. coli*, cross-reacted.

FHA and MOMP were the top matches in protein identification from gel bands in the 220 and 25 kDa regions, respectively, using LC MS/MS protein identification. However, the recombinant MOMP expressed in *E. coli* did not show any immunogenicity on western blots probed using SLD positive sera, probably indicating that the immunogenic protein within the 25 kDa region is a less abundant protein of similar size to MOMP. The finding of FHA and MOMP in both LC MS/MS protein identification and bioinformatics analysis also supports the rationale used in the applied molecular and bioinformatics analysis methods to identify a candidate protein for ELISA development.

Furthermore, FHA_1,628−1,899_ is 100% conserved among 12 Victorian and South Australian *C. hepaticus* strains and 96% conserved among the two Queensland strains (2). FHA_1,628−1,899_ also shared 98.55% identity with the USA *C. hepaticus* strain (40) and 97.80% identity with the UK *C. hepaticus* strains (7). The high similarity of FHA_1,628−1,899_ among *C. hepaticus* strains isolated from geographically distinct locations suggests the universal application of this assay. Another species of campylobacter named as *Campylobacter bilis*, has recently been isolated from birds with SLD in Australia (41). However, the homologous FHA fragment in *C. bilis* only share 56% amino acid identity with FHA_1,628−1,899_. The effectiveness of SLD-ELISA2 in detecting anti-FHA_1,628−1,899_ antibodies in *C. bilis* infected birds needs to be experimentally determined.

The specificity and sensitivity of SLD-ELISA2, 95 and 93%, respectively, were slightly lower than that of SLD-ELISA1 for experimentally infected and negative control samples. However, SLD-ELISA2 detected anti-FHA_1,628−1,899_ antibodies in all 10 naturally infected samples. Three samples in the negative control group were positive to SLD-ELISA2 suggestive of them being true positives with high anti-FHA_1,628−1,899_ antibody titer that was not detected by SLD-ELISA1, or the presence of antibodies cross-reacting with FHA_1,628−1,899_ in the bird sera. The experimentally infected group consisted of birds 3, 6, 9, and 12-weeks post *C. hepaticus* challenge and sera from most birds had antibodies against FHA_1,628−1,899_. The serum from one bird tested negative to both ELISAs, indicative of the lack of SLD specific immune response in that bird. Three other birds in the experimentally infected group had anti-FHA_1,628−1,899_ antibody levels slightly lower than the assay cut-off value. However, SLD-ELISA1 was able to detect *C. hepaticus* specific antibodies from these samples because the absorbance obtained in SLD-ELISA1 is a collective response from the antibody cohort generated against the entire collection of *C. hepaticus* proteins whereas SLD-ELISA2 measures the antibodies that specifically recognize the immunogenic fragment of FHA. The bird-to-bird variation in antibody titres could be another reason for the slightly higher assay sensitivity for SLD-ELISA1. Nevertheless, considering the extra resources and time required for performing the pre-absorption step in SLD-ELISA1, SLD-ELISA2 is a more convenient option, especially when working with large sample sizes as in the identification of the seroconverted birds in farms or in assessing the immune response of birds in large scale experimental infection studies. SLD-ELISA2 has been used to assay sera samples collected from more than 700 birds in commercial free-range farms to better understand the seroprevalence of anti-*C. hepaticus* antibodies in Australian free-range farms (42).

The SLD-ELISA2 performance/accuracy was assessed based on intra and inter assay precision/ variation calculated as %CV. The intra-assay variation (between-run) was 13.7% and the inter-assay variation (within run) ranged between 1 and 9%. Both inter and intra-assay precision was within the acceptable limits of 15% (25) and demonstrates the reliability and repeatability of the assay.

To conclude, this study has identified a *C. hepaticus* protein, filamentous hemagglutinin adhesin, and has developed it for use as a capture ligand to identify *C. hepaticus* specific antibodies. The newly developed ELISA could detect birds that has previously been exposed to *C. hepaticus* infection and can therefore be used in SLD epidemiological studies. The main finding of the study, *C. hepaticus* specific antigenicity of FHA, has also opened opportunities for future research in SLD vaccine and pathogenicity studies.

## Data availability statement

The original contributions presented in the study are included in the article/supplementary material, further inquiries can be directed to the corresponding author.

## Ethics statement

The animal study was reviewed and approved by Agriculture Victoria, Wildlife and Small Institutions Animal Ethics Committee (Approval number 33.21).

## Author contributions

TV and RM conceived the study and edited the manuscript. CM designed and performed the experiments. JQ, AA, TW, and PS collected the samples and arranged for sample collection by other veterinarians. CM drafted the manuscript. All authors have edited and approved the final manuscript.
